# In Silico Systems Biology Approach for Prioritization of Candidate Genes Linked to Lipid Metabolism in the Context of Cardiovascular Disease Susceptibility in a Serbian Cohort

**DOI:** 10.3390/cimb48060613

**Published:** 2026-06-12

**Authors:** Tamara Drljača, Vladimir Perović, Nevena Veljković, Branislava Gemović

**Affiliations:** Laboratory for Bioinformatics and Computational Chemistry, Institute of Nuclear Sciences Vinča, National Institute of the Republic of Serbia, University of Belgrade, 11000 Belgrade, Serbia; tamara.drljaca@vin.bg.ac.rs (T.D.);

**Keywords:** cardiovascular diseases, lipid metabolism, computational biology, *PSPH*, variant combinations

## Abstract

Background: The population of Serbia faces a significant burden from cardiovascular diseases (CVDs). This study aimed to computationally investigate genetic factors that contribute to the prevalence of these diseases by examining the possible involvement of common variants on lipid metabolism. Methods: We examined how a variant prevalent in the Serbian population, chr7:g.56019730G>A in the *PSPH* gene, affects the phosphoserine phosphatase (PSP) protein interaction network, particularly involved in lipid metabolism. The Informational Spectrum Method (ISM), method for the analysis of protein sequence based on amplitude changes, was applied to single out the top 10 affected interactors. Their further functional annotation identified the pathways in which they jointly participate with PSP. An additional strategy encompassed the investigation of variant combinations in all analyzed genes and potential relevance of prevalent variant combinations on lipid metabolism. Results: The PSP interactions affected by the R49W variant, such as SHMT1/2, were primarily in pathways associated with serine, glycine, and sphingolipid metabolism, highly relevant for CVD etiology. Further, we identified frequent variant combinations within the *LRCH1*, *CEP126*, *PIK3CG,* and *PIKFYVE* genes in the Serbian cohort. Conclusions: This study underscores the importance of investigating genetic variant combinations in complex diseases, and provides a hypothesis generating foundation for future research into the relationship between these genes and cardiovascular diseases.

## 1. Introduction

Non-communicable complex diseases, like cancers, diabetes, and cardiovascular diseases (CVDs), take 41 million lives yearly, with CVDs accounting for the most deaths, totaling 17.9 million people annually [1]. Due to their high prevalence, complex diseases place a significant burden on both the human population and healthcare and economic systems. Early detection and identification of risk factors contributing to complex diseases are crucial for mitigating their impact [2].

Massive parallel sequencing, complemented by bioinformatics analyses, is now used to detect genetic variants associated with rare and common diseases [3,4]. Together, these methods have enhanced the investigation process of genetic diseases and the search for effective treatments; however, with the advancement of technology, shortcomings in diagnostics have been noted in the form of a lack of reference panels for underrepresented subpopulations. The genetic patterns of diverse subpopulations are becoming more visible, and the need for population-relevant biomarkers is becoming evident [5].

The population of Serbia is heavily burdened with various non-communicable diseases, with CVDs being the leading cause of death, making up 49.8% of all deaths according to 2023 data [6]. From 2014 to 2023, death rates from diseases caused by hypertension have increased in Serbia by 103.5%, with acute coronary syndrome (ACS) accounting for 49% of all deaths from ischemic heart disease in 2023 [6]. Another leading non-communicable disease cause of death is T2 diabetes mellitus, a common comorbidity, affecting 8.1% of the adult population [6]. According to the National Health Survey from 2019, in Serbia, 57.1% of the population were overweight, among which 36.3% were pre-obese and 20.8% were obese [7]. Metabolic syndrome refers to a collection of risk factors linked to obesity that increase the likelihood of CVDs [8]. A key emerging risk factor within this syndrome is dyslipidemia, characterized by elevated triglycerides and low-density lipoprotein cholesterol (LDL), alongside reduced high-density lipoprotein cholesterol (HDL) [9,10]. Moreover, a recent large-scale study showed that lipidomic profiles, including different lipid species beyond traditional lipids analyzed in dyslipidemia, capture more information and suggest that previously unconsidered lipid species can also enhance the risk of CVDs [11]. Additionally, the study found that the plasma levels of these lipid species have heritable traits, with varying degrees of heritability [11].

In this study, we applied in silico analyses to investigate associations between genetic patterns observed in the Serbian cohort and alterations in lipid metabolism potentially contributing to dysregulated lipid metabolism associated with cardiovascular disease. By analyzing the systemic effects of common variants, we aimed to prioritize candidate genes with unexplored relevance to CVD, which may, through specific protein interactions, contribute to the dysregulation of lipid metabolism. The genetic structure of the population of modern Serbia was analyzed using clinical exome next-generation sequencing data focusing on common variants, revealing certain features when compared to other European populations [12]. Furthermore, a missense variant in the *PSPH* gene (chr7:g.56019730G>A), causing an R49W amino acid substitution, was identified as frequent in the Serbian population sample (MAF = 0.163), with functional impact predicted by SIFT and MutPred2 [12]. The Serbian cohort from the previous study [12] served as the discovery dataset for identifying candidate genes for further computational investigation. Variants and variant combinations were selected based on their observed frequency in the cohort and predicted functional impact and were subsequently investigated for their predicted involvement in lipid metabolism pathways. We explored potential mechanisms computationally linking these variants to lipid metabolism and CVDs, while prioritizing candidate genes for further investigation based on the frequency and predicted functional impact of variant combinations observed in the Serbian cohort. Given the established role of *PSPH* in serine biosynthesis and emerging evidence linking serine metabolism to cardiovascular pathways, this variant was selected for further computational investigation.

We hypothesize that the R49W variant of phosphoserine phosphatase (PSP), encoded by the *PSPH* gene may alter interactions between PSP and its protein interactors, potentially affecting serine and glycine metabolism. Given the established roles of these amino acids in sphingolipid and glycerophospholipid biosynthesis, such disruption may contribute to dyslipidemia and metabolic syndrome, potentially underlying the elevated burden of cardiovascular diseases observed in Serbia. This study aims to computationally explore and support this hypothesis as a foundation for future experimental investigation.

## 2. Materials and Methods

The dataset that was used in this study consists of genetic variants found in a sample of the population of Serbia after analyzing the clinical exome of this population [12].

To determine the presence of variants potentially involved in the occurrence of CVDs in the population of Serbia, we analyzed the clinical exome of the sample of the population of Serbia. The dataset analyzed in this research contains a cohort of 144 samples from the population of Serbia. Samples were sequenced using target exome sequencing with the Illumina TruSight One kit, and the data were processed using the GATK variant calling protocol. A detailed process of pre-processing clinical exome data and variant calling was previously described in Drljaca et al. [12]. Sequencing data were mapped to the GRCh38 version of the human reference genome. The dataset that was used in this study is openly available in European Variation Archive at https://www.ebi.ac.uk/eva/?eva-study=PRJEB42044, accessed on 30 May 2026, reference number PRJEB42044.

Further investigation into the genetic variant chr7:g.56019730G>A in the *PSPH* gene, with a minor allele frequency of 0.163 in the Serbian population sample [12], focused on its impact on the phosphoserine phosphatase (PSP) protein and analyzed its interactions. We computationally investigated whether the R49W amino acid change affects the interaction between the PSP protein and its interactors using the Informational Spectrum Method (ISM) [13]. The goal was to computationally evaluate whether there is a change in the ISM signal between PSP and its interactors due to the presence of the R49W amino acid change, and whether such calculated changes in signal may be relevant to lipid metabolism pathways. This analysis consists of (1) the retrieval of PSP interactors, (2) ISM protocol, and (3) functional annotation of ISM-selected interactors and PSP.

PSP interactors were obtained from the STRING database [14]. The parameters used to search the STRING database were: interaction source: databases, experimentally proven; maximum number of interactors shown: 100.

The Informational Spectrum Method (ISM) [13] is a method for the analysis of protein sequences. ISM has been applied to a range of protein interaction problems, including the analysis of SARS-CoV-2 spike protein interactions and therapeutic target identification [15] and independent computational approaches based on similar Fourier transform principles have demonstrated effective PPI prediction across multiple organisms [16]. In the first step, the protein sequence is transformed into a vector of numbers by assigning each amino acid its Electron–Ion Interaction Potential (EIIP) (Supplementary Table S1) [17].

In the second step, the EIIP numerical sequence is then subjected to discrete Fourier transformation defined as follows:

(1) X(n) = ∑_m=1…n_ x(m)e^−i2πnm/N^, n = 1…N/2

where x(m) is the m-th member of an EIIP numerical series, N is the total number of points in the series, and X(n) is the discrete Fourier transformation (DFT) coefficient.

The absolute value of the complex Fourier transformation defines the amplitude spectrum and the phase spectrum, where in the case of protein analysis information is represented as an energy density spectrum, defined as

(2) S(n) = X(n)X × (n) = |X(n)|^2^, n = 1…N/2

The generated Informational Spectrum (IS) is given as the series of frequencies and corresponding amplitudes that represent the analyzed protein. The primary structures of interacting proteins encode the common information which is represented by the same code/frequency pair(s) in their ISs. Cross-spectrum or Consensus Informational Spectrum (CIS) determines this common informational characteristic of sequences.

The following equation calculates the CIS of two ISs:

(3) C(i) = S_1_(i) S_2_(i), I = 1…N/2

where S_1_(i) and S_2_(i) are the i-th elements of the first and second IS correspondingly, and C(i) is the i-th element of CIS. Peak frequencies in CIS represent common information in analyzed proteins and they are characterized by the amplitude and the signal-to-noise ratio (S/N), i.e., the ratio of the amplitude value on a particular frequency and the sum of amplitudes on all frequencies in IS.

**Figure 1:**
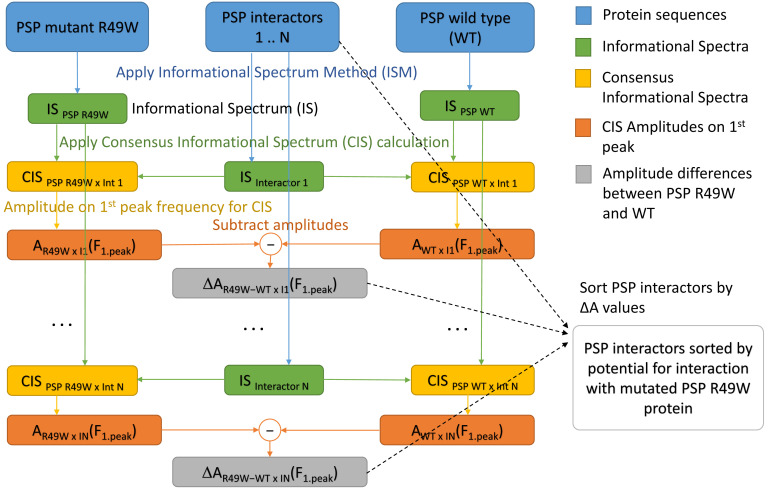
ISM protocol—A schematic view of the ISM-based analysis. For the input sequences, PSP wildtype and R49W mutant, and PSP interactors (blue), their ISs are generated (green), as well as the CIS of the PSP and each of its interactors (yellow). The CIS amplitudes on the first peak frequency are selected (orange), and the differences between PSP wildtype and R49W mutant (gray) for each CIS are calculated and used to sort the interactors by the potential for interaction.

For the analysis of the impact of the R49W mutation on the ISM amplitude signal, we performed the following procedure (Figure 1). For each interactor of the PSP protein, the CIS of the interactor and PSP wildtype is calculated. The same step is repeated for the R49W PSP mutant. Then, the delta values of the amplitudes (S/Ns) on the first peak in each generated CIS between the PSP wildtype and the mutant are calculated, and the interactors are sorted by those values. In this way, the top interactors have the largest change in amplitude on the first peak of the CIS when the mutation is introduced, thus indicating the most increased potential for interaction with the mutated PSP protein. Similarly, the interactors with the smallest change, i.e., the largest negative change, have the most decreased potential for interaction.

To explore which pathways and biological processes may be computationally linked to the predicted changes in PSP interactions caused by the R49W variant, we analyzed what pathways and biological processes PSP and its ISM-selected interactors engage in. After the application of ISM, we created a subset of 10 PSP interactors by extracting interactors with the highest change in ISM amplitude: five interactors with the highest and five interactors with the lowest ISM amplitude after the introduction of the R49W amino acid change. Enrichment analysis was applied to the ISM-selected interactors and PSP using the DAVID tool v6.8 [18], as well as annotation on the Kyoto Encyclopedia of Genes and Genomes (KEGG) pathways [19], and Gene Ontology (GO), with the biological processes (BP) subontology [20,21]. The background gene set used was all genes for *Homo sapiens* in DAVID.

To computationally predict candidate genes that could contribute to the occurrence of CVDs, according to the observations in the Serbian cohort, we investigated whether there are highly frequent variant combinations involved in lipid metabolism in our sample. The sample was restricted to common variants with a minor allele frequency of 5% for this analysis. The number of combinations was restricted to seven. Each combination contains the variants from the same gene. The percentage of occurrence for the combination of variants is calculated so that the presence of the combination in a sample is true if and only if all variants are present in the sample. Annotation of genes with common variant combinations in DAVID for KEGG pathways and GO BP was done to explore whether any of these genes show predicted associations with lipidomic pathways.

Additionally, to assess whether the identified variant combinations reflect independent variants or common haplotypic structure, we checked pairwise linkage disequilibrium (LD) of these variant combinations, for each gene separately. LD was done using vcftools (v0.1.16) [22] with the following parameters: --ld-window-bp 500,000 –ld-window-bp-min 1 –max-alleles 2 –min-alleles 2 –min-r^2 0 –geno-r2. To verify whether the variant pairs observed in perfect LD within the Serbian cohort exhibit consistent LD patterns in broader European reference populations, pairwise linkage disequilibrium analyses were performed using the LDpop tool from the LDlink suite (https://ldlink.nih.gov/ldpop, accessed on 28 May 2026) [23,24]. Representative variant pair from the *PIKFYVE* seven variant combination was selected for analysis, prioritizing the most spatially distant pair to capture the extent of the haplotype block. LD statistics (r^2^) was retrieved for European reference populations available in the 1000 Genomes Project Phase 3 [25], including the subpopulations of Utah residents from Central Europe (CEU), Toscani in Italy (TSI), Finnish in Finland (FIN), British in England (GBR) and Iberian population in Spain (IBS).

## 3. Results and Discussion

### 3.1. Annotation of PSP Interactors

In this study, we explored the possible contribution that the effect of chr7:g.56019730G>A, a *PSPH* gene variant, could have to CVD-related pathways in the cohort of Serbia by analyzing the potential impact that the R49W change has on the PSP interactions. After applying the ISM on 38 experimentally confirmed PSP interactors (Supplementary Table S2), we singled out a subset of 10 interactors (Table 1) with the highest predicted change in ISM amplitude in the cross-spectrum after introducing the R49W amino acid change.

We investigated the biological processes and signaling pathways involving PSP and the interactors predicted to be the most affected by the R49W variant in order to determine the physiological context in which this variant may exert the strongest impact. Table 2 shows the predicted signaling pathways in which ISM-selected PSP interactors and PSP are involved together. Most of the predicted pathways relate to serine metabolism, which is expected, as the proteins involved in these pathways are PSP interactors and are catalysts of L-serine biosynthesis. In silico analysis of the KEGG pathway shows that PSP is found together with SHMT1 and SHMT2 in glycine, serine, and threonine metabolism and in the biosynthesis of amino acids, as well as in carbon metabolism (Table 2).

GO BP results also show that the presence of these interactors is predicted in serine metabolic and biosynthetic processes (Table 3). According to the prior literature, PSP catalyzes the last irreversible step in the biosynthesis of L-serine from carbohydrates [26]. SHMT catalyzes the transfer of the hydroxymethyl group from L-serine to tetrahydrofolate to yield glycine and 5–10 methylenetetrahydrofolate in a reversible reaction [27]. SHMT1 is a cytosolic isoform, while SHMT2 is a mitochondrial isoform. Considering the role of these proteins in serine and glycine metabolism and biosynthesis, we hypothesize that the in silico calculated change in interaction between these proteins, as a consequence of the R49W variant in PSP, may be relevant to serine and glycine level regulation, though this remains to be experimentally validated.

Serine, as the central metabolite that connects the signaling pathways and biological processes in which PSP interactors are involved, is a non-essential amino acid involved in a number of biochemical and molecular mechanisms, with a variety of roles [28]. From what is previously known serine is relevant for CVD risk. As found in multiple CVD-related pathways, L-serine is described as a potential biomarker candidate for CVDs [29]. Furthermore, serine can be connected with potential involvement in hypertension, considering that direct blood pressure-lowering effects of serine are reported [30], which implies a potential alteration in blood pressure control in the case of lowering the serine level [29]. A meta-analysis of patient data in which 10 new metabolites were associated with myocardial infarction showed that glyoxylate and dicarboxylate metabolism and glycine, serine, and threonine metabolism are significantly associated with incident myocardial infarction [31]. The role of amino acid levels in dyslipidemia, such as serine and glycine, is becoming clearer. The study consisting of the Japanese population showed that levels of serine were significantly related to the development of metabolic syndrome, while the level of glycine was related to developing dyslipidemia [32]. Similarly, the study with Chinese patients concluded that serine might play a protective role in coronary heart disease [33]. Taken together, conclusions from the literature support the biological plausibility of our computational hypothesis that R49W-induced changes in PSP interactions could, if experimentally confirmed, have downstream relevance to CVD risk.

Serine also has an important role in lipid metabolism, as it is one of the two substrates for de novo ceramide synthesis, which is one of the biologically active sphingolipids [34]. Our computational analysis predicts that interactors of the PSP are also present in the lipid metabolic process (Table 3). Based on the prior literature, the role of sphingolipids, as a class of lipids, in the pathophysiology of CVDs is starting to be clearer. Recent studies show the role of sphingolipids in the pathophysiology of hypertension, as well as their role in arterial calcification and atherosclerosis in general, that is, the occurrence of coronary artery disease [35]. Furthermore, ceramides have a role in the infiltration of LDL in blood vessels and its aggregation, which further leads to atherosclerotic processes and CVDs [36]. The role of glycerophospholipid metabolism in the pathophysiology of CVDs has wide potential for further exploration, considering that the metabolites involved in this path have a role in coronary artery disease progression [37]. Additionally, the study on mice showed that the disturbance in sphingolipid and glycerophospholipid metabolism might indicate atherosclerotic progression [38].

### 3.2. Overrepresented Variant Combinations Involved in Lipid Metabolism

To identify potentially novel candidate genes in the Serbian cohort, we analyzed highly frequent co-occurring variants without restricting the analysis to genes previously associated with cardiovascular diseases or lipid metabolism. Our algorithm extracted variant combinations occurring within the same gene (Supplementary Data), with the highest occurrence (>95%) identified in three genes (Table 4). PIKFYVE was the only gene in which combinations of up to seven variants were observed at a frequency above 90% (Table 5).

Further analysis was conducted on the four genes *LRCH1*, *PIK3CG*, *CEP126*, and *PIKFYVE*. To assess whether the identified variant combinations reflect independent mutational events or common haplotypic structure, pairwise LD analysis was performed for each gene separately. The results revealed that the variant combinations identified in *LRCH1*, *PIK3CG*, *CEP126*, and *PIKFYVE* are in perfect or near-perfect linkage disequilibrium (r^2^ = 1.0) (Supplementary Figure S1), indicating that these combinations likely represent common haplotypic blocks rather than independently occurring variants. This finding is consistent with the established population genetic principle that variation in human population is structured into haplotypes transmitted as units [39].

To verify whether the variant pairs observed in perfect LD within the Serbian cohort exhibit consistent LD patterns in broader European reference populations, pairwise LD analysis of LD patterns across European 1000 Genomes Project [25] populations was performed using the LDpop tool from the LDlink suite [23,24]. The results (Supplementary Table S3) confirmed that variant pairs from all four genes are in perfect or near-perfect LD across European populations. Specifically, variant pairs in *CEP126* showed r^2^ = 1.0 across all European subpopulations examined, representing the most consistent pattern. Variant pairs in *PIK3CG* showed r^2^ = 1.0 in most subpopulations, with slightly lower but still high values in the broader EUR superpopulation (r^2^ = 0.977) and GBR (r^2^ = 0.884), likely reflecting minor allele frequency differences across subpopulations rather than genuine LD breakdown. For *LRCH1* and *PIKFYVE*, r^2^ = 1.0 was confirmed in all subpopulations where the minor allele was present. Taken together, these results indicate that the haplotypic structures observed in the Serbian cohort reflect conserved European haplotype blocks, consistent with the shared demographic history of European populations [40].

While this finding limits the ability to attribute functional effects to individual variants within each combination, it does not preclude the possibility that the haplotype may be functionally relevant [41]. In the context of complex diseases, where cumulative effects of common variants are increasingly recognized, such haplotypic structures may themselves represent biologically relevant units for future investigation [41].

The complete enrichment annotation results are provided in Supplementary Tables S4 and S5 for transparency and should be interpreted as exploratory observations only. Although formal enrichment analysis was underpowered due to the small gene set size, the existing literature supports the potential relevance of *PIK3CG*, *PIKFYVE*, and *LRCH1* to lipid metabolism-related pathways, as discussed below. The functional relevance of *CEP126* in this context remains to be established. Phosphatidylinositol-3-phosphate (PI3P) plays a significant role in various cellular processes, including signaling pathways that are crucial for lipid metabolism and homeostasis. It has been shown that impaired PI3P signaling leads to metabolic disorders and plays a role in the development of cardiac pathophysiology [42]. Furthermore, peripheral insulin resistance has been suggested to be the product of impaired PI3K signaling in the effector cells. Insulin resistance is highly connected with metabolic syndrome, and its presence often includes dyslipidemia [8].

PIK3CG is part of the class I phosphoinositide 3-kinases and is primarily activated by G protein-coupled receptors (GPCRs). When activated, PIK3CG phosphorylates phosphatidylinositol 4,5-bisphosphate (PIP2) to produce PIP3. The literature shows that PIK3CG is also involved in lipid metabolism. It has been shown that common genetic variation in the *PIK3CG* locus determines plasma HDL-cholesterol concentrations [43]. Furthermore, *PIK3CG* variants are associated with vascular calcification [44].

PIKFYVE specifically phosphorylates PI3P at the five-position of the inositol ring, generating PI(3,5)P2. This conversion is crucial for maintaining the balance of phosphoinositides in cellular membranes. By converting PI3P to PI(3,5)P2, PIKFYVE helps regulate the levels of different phosphoinositides, which is essential for maintaining cellular signaling and membrane integrity [45]. Furthermore, PIKfyve depletion in platelets has been associated with defective lysosomal maturation, inflammation and thrombosis in mouse models, suggesting a potential role in cardiovascular relevant cellular processes [45].

Although the combination of variants in the *LRCH1* gene found at great frequency (Table 4) was not reported in the ClinVar database [46], the literature shows that this gene might be interesting in the context of CVD development. The *LRCH1* gene encodes a protein that is involved in various cellular processes, including the regulation of immune responses and cell signaling [47]. Transcriptome-wide association studies showed that *LRCH1* is involved in the mechanism of stroke and can be considered a risk gene [48]. Furthermore, in other studies, the *LRCH1* gene was identified as significant for platelets, systolic blood pressure, and stroke [49]. *LRCH1* was also identified as a candidate in the mechanism of atherosclerosis [50]. Although there is yet no direct evidence of the role that *LRCH1* has in dyslipidemia, the previously mentioned studies indicate that this potential role might be possible.

### 3.3. Limitations and Future Work

The findings of this study should be interpreted in the context of its computational, hypothesis-generating design focused on the Serbian cohort. Several limitations should therefore be considered when interpreting the biological and population-level relevance of the identified variants and predicted interactions.

In future work, a larger population sample would be needed to fully explore the levels of population specificity in the context of lipid metabolism dysregulation. Furthermore, experimental confirmation of altered protein interaction, would be needed to complement these results. Additionally, allele frequency estimates for variants identified in this study should be interpreted in the context of evolving reference databases, as improved sequencing coverage in updated reference datasets may refine population frequency comparisons.

Further functional validation of the *PSPH* gene variant is needed to support the computational predictions presented in this study. Future studies should experimentally investigate whether the R49W variant alters interactions between PSP and SHMT1/2 proteins and whether such changes affect serine and glycine metabolism. In addition, metabolomic profiling would be required to assess potential alterations in serine and glycine levels, while lipidomic analyses in relevant cell models could help determine whether these metabolic changes are associated with lipid metabolism dysregulation. Ideally, these findings should be further evaluated in a larger Serbian cohort with available lipid-related clinical measurements.

Regarding the variant combinations identified in *LRCH1*, *PIK3CG*, *CEP126*, and *PIKFYVE*, further research is needed to determine whether these haplotypes are of functional significance regarding relevance to CVD susceptibility.

## 4. Conclusions

This study computationally predicts that the R49W variant of phosphoserine phosphatase (PSP), encoded by the *PSPH* gene, may alter interactions with SHMT1 and SHMT2 proteins, with potential downstream relevance to serine and glycine metabolism and associated lipid metabolism pathways. Although these findings remain to be experimentally validated, they support the biological plausibility of a potential link between *PSPH*-related metabolic alterations and cardiovascular disease-associated pathways.

Furthermore, this study identifies *LRCH1, PIKFYVE*, and *PIK3CG* as candidate genes for future investigation of their potential roles in lipid metabolism-related processes and cardiovascular disease risk based on in silico analyses and population-level variant patterns observed in the Serbian cohort. The identified high-frequency variant combinations were found to form strong haplotypic blocks, suggesting stable co-inheritance patterns that may be relevant for future investigation in the context of complex disease risk.

Overall, these findings highlight the potential value of investigating common variants and haplotypic structures in less-characterized European populations for the computational prioritization of novel candidate genes relevant to complex diseases.

## Figures and Tables

**Table 1:** ISM-selected interactors subset. To analyze in silico whether the R49W variant will affect change to the interaction of PSP, we applied the ISM protocol to PSP interactors. The table shows PSP interactors that, with the introduction of R49W, have the highest and lowest ISM amplitude change. The subset of 10 interactors with the highest amplitude change is referred to as ISM-selected interactors.

Uniprot Accession Number	Gene Name	Amp(R49W) − Amp(WT)
Q9UH90	*FBXO40*	0.297
O14939	*PLD2*	0.257
Q32NB8	*PGS1*	0.239
Q8IYQ7	*THNSL1*	0.233
P34896	*SHMT1*	0.209
P14324	*FDPS*	−0.091
P34897	*SHMT2*	−0.205
Q9NUV7	*SPTLC3*	−0.235
Q86YJ6	*THNSL2*	−0.256
Q8N2Y8	*RUSC2*	−0.816

**Table 2:** KEGG predicted pathways in which ISM-selected interactors and PSP are involved together. KEGG pathway annotation was applied through the utilization of the DAVID software in order to distinguish the predicted pathways in which ISM-selected interactors and PSP occur together.

Term	The Genes Which Encode an Interactor	*p*-Value	False Discovery Rate (FDR)
Metabolic pathways	farnesyl diphosphate synthase (*FDPS*);	2.13 × 10^−5^	6.17 × 10^−4^
	phosphatidylglycerophosphate synthase 1 (*PGS1*);		
	phospholipase D2 (*PLD2*);		
	phosphoserine phosphatase (*PSPH*);		
	serine hydroxymethyltransferase 1 (*SHMT1*);		
	serine hydroxymethyltransferase 2 (*SHMT2*);		
	serine palmitoyltransferase long chain base subunit 3 (*SPTLC3*)		
Glycine, serine and threonine metabolism	serine hydroxymethyltransferase 1 (*SHMT1*);	2.70 × 10^−4^	3.91 × 10^−3^
	serine hydroxymethyltransferase 2 (*SHMT2*);		
	phosphoserine phosphatase (*PSPH*)		
Biosynthesis of amino acids	serine hydroxymethyltransferase 1 (*SHMT1*);	9.05 × 10^−4^	8.74 × 10^−3^
	serine hydroxymethyltransferase 2 (*SHMT2*);		
	phosphoserine phosphatase (*PSPH*)		
Carbon metabolism	serine hydroxymethyltransferase 1 (*SHMT1*);	2.19 × 10^−3^	1.58 × 10^−2^
	serine hydroxymethyltransferase 2 (*SHMT2*);		
	phosphoserine phosphatase (*PSPH*)		

**Table 3:** Gene Ontology biological processes in which ISM-selected PSP interactors and PSP are predicted to be involved. This annotation was conducted by utilizing DAVID software.

Term	Genes	*p*-Value	False Discovery Rate (FDR)
L-serine metabolic process	serine hydroxymethyltransferase 1 (*SHMT1*);	6.61 × 10^−6^	3.97 × 10^−4^
	serine hydroxymethyltransferase 2 (*SHMT2*);		
	phosphoserine phosphatase (*PSPH*)		
Glycine biosynthetic process from serine	serine hydroxymethyltransferase 1 (*SHMT1*);	8.20 × 10^−4^	2.46 × 10^−2^
	serine hydroxymethyltransferase 2 (*SHMT2*)		
L-serine biosynthetic process	phosphoserine phosphatase (*PSPH*);	2.46 × 10^−3^	4.50 × 10^−2^
	serine hydroxymethyltransferase 2 (*SHMT2*)		
Glycine metabolic process	serine hydroxymethyltransferase 1 (*SHMT1*);	3.28 × 10^−3^	4.50 × 10^−2^
	serine hydroxymethyltransferase 2 (*SHMT2*)		
Lipid metabolic process	farnesyl diphosphate synthase (*FDPS*);	3.87 × 10^−3^	4.50 × 10^−2^
	phosphatidylglycerophosphate synthase 1 (*PGS1*);		
	serine palmitoyltransferase long chain base subunit 3 (*SPTLC3*)		
	phospholipase D2 (*PLD2*);		
Tetrahydrofolate interconversion	hydroxymethyltransferase 1(*SHMT1*);	4.50 × 10^−3^	4.50 × 10^−2^
	serine hydroxymethyltransferase 2 (*SHMT2*);		
Tetrahydrofolate metabolic process	hydroxymethyltransferase 1 (*SHMT1*);	5.32 × 10^−3^	4.56 × 10^−2^
	serine hydroxymethyltransferase 2 (*SHMT2*);		

**Table 4:** Variant combination with the highest occurrence in the sample. Variant combinations found in the same gene at the highest frequency after applying the algorithm to find overrepresented variant combinations in the sample of the Serbian cohort.

Gene	Variant Combinations	% of Occurrence in the Sample
*LRCH1*	chr13:g.46733974A>G; chr13:g.46741849T>C	0.965278
*PIK3CG*	chr7:g.106868533A>G; chr7:g.106868542T>C	0.958333
*CEP126*	chr11:g.101962913C>T; chr11:g.101987027G>A	0.958333

**Table 5:** Variant combination with the highest number of variants. Gene *PIKFYVE* was the only gene found with the highest number of combinations (7) at this high rate above 90% after applying the algorithm to find overrepresented variant combinations in the sample of the Serbian cohort.

Gene	Variant Combinations	% of Occurrence in the Sample
*PIKFYVE*	chr2:g.208320275C>T; chr2:g.208325795A>T; chr2:g.208325804C>G; chr2:g.208345103G>A; chr2:g.208350046A>G; chr2:g.208350862A>G; chr2:g.208325606T>C	0.9375

## Data Availability

The data that supports the findings of this study are openly available in European Variation Archive at https://www.ebi.ac.uk/eva/?eva-study=PRJEB42044, accessed on 30 May 2026, reference number PRJEB42044.

## Supplementary Materials

The following supporting information can be downloaded at: https://www.mdpi.com/article/10.3390/cimb48060613/s1.

## References

[1] World Health Organization https://www.who.int/news-room/fact-sheets/detail/noncommunicable-diseases#:~:text=Key%20facts,%2D%20and%20middle%2Dincome%20countries.

[2] Karunathilake S.P., Ganegoda G.U. (2018). Secondary Prevention of Cardiovascular Diseases and Application of Technology for Early Diagnosis. BioMed Res. Int..

[3] Yang Y., Muzny D.M., Reid J.G., Bainbridge M.N., Willis A., Ward P.A., Braxton A., Beuten J., Xia F., Niu Z. (2013). Clinical Whole-Exome Sequencing for the Diagnosis of Mendelian Disorders. N. Engl. J. Med..

[4] Papadopoulou E., Bouzarelou D., Tsaousis G., Papathanasiou A., Vogiatzi G., Vlachopoulos C., Miliou A., Papachristou P., Prappa E., Servos G. (2023). Application of next generation sequencing in cardiology: Current and future precision medicine implications. Front. Cardiovasc. Med..

[5] Schmitt T., Poirel H.A., Cauët E., Delnord M., Van Den Bulcke M. (2024). Unlocking the genomic landscape: Results of the Beyond 1 Million Genomes (B1MG) pilot in Belgium towards Genomic Data Infrastructure (GDI). Health Policy.

[6] Institute for Public Health of Serbia “Dr Milan Jovanovic Batut” (2024). Health Statistical Yearbook of Republic of Serbia 2023.

[7] National Health Survey 2019. https://publikacije.stat.gov.rs/G2021/pdfE/G20216003.pdf.

[8] Grundy S.M. (2004). Obesity, Metabolic Syndrome, and Cardiovascular Disease. J. Clin. Endocrinol. Metab..

[9] Abera A., Worede A., Hirigo A.T., Alemayehu R., Ambachew S. (2024). Dyslipidemia and associated factors among adult cardiac patients: A hospital-based comparative cross-sectional study. Eur. J. Med. Res..

[10] Hedayatnia M., Asadi Z., Zare-Feyzabadi R., Yaghooti-Khorasani M., Ghazizadeh H., Ghaffarian-Zirak R., Nosrati-Tirkani A., Mohammadi-Bajgiran M., Rohban M., Sadabadi F. (2020). Dyslipidemia and cardiovascular disease risk among the MASHAD study population. Lipids Health Dis..

[11] Tabassum R., Project F., Rämö J.T., Ripatti P., Koskela J., Kurki M., Karjalainen J., Palta P., Hassan S., Nunez-Fontarnau J. (2019). Genetic architecture of human plasma lipidome and its link to cardiovascular disease. Nat. Commun..

[12] Drljaca T., Zukic B., Kovacevic V., Gemovic B., Klaassen-Ljubicic K., Perovic V., Lazarevic M., Pavlovic S., Veljkovic N. (2021). The first insight into the genetic structure of the population of modern Serbia. Sci. Rep..

[13] Veljkovic V., Cosic I., Dimitrijevic B., Lalovic D. (1985). Is it Possible to Analyze DNA and Protein Sequences by the Methods of Digital Signal Processing?. IEEE Trans. Biomed. Eng..

[14] Szklarczyk D., Kirsch R., Koutrouli M., Nastou K., Mehryary F., Hachilif R., Gable A.L., Fang T., Doncheva N.T., Pyysalo S. (2023). The STRING database in 2023: Protein-protein association networks and functional enrichment analyses for any sequenced genome of interest. Nucleic Acids Res..

[15] Perovic V., Glisic S., Veljkovic M., Paessler S., Veljkovic V. (2024). In Silico Exploration of CD200 as a therapeutic target for COVID-19. Microorganisms.

[16] Yin C., Yau S.S.-T. (2017). A coevolution analysis for identifying protein-protein interactions by Fourier transform. PLoS ONE.

[17] Veljkovic V., Slavic I. (1972). Simple General-Model Pseudopotential. Phys. Rev. Lett..

[18] Sherman B.T., Hao M., Qiu J., Jiao X., Baseler M.W., Lane H.C., Imamichi T., Chang W. (2022). DAVID: A web server for functional enrichment analysis and functional annotation of gene lists (2021 update). Nucleic Acids Res..

[19] Kanehisa M. (2000). KEGG: Kyoto Encyclopedia of Genes and Genomes. Nucleic Acids Res..

[20] Ashburner M., Ball C.A., Blake J.A., Botstein D., Butler H., Cherry J.M., Davis A.P., Dolinski K., Dwight S.S., Eppig J.T. (2000). Gene Ontology: Tool for the unification of biology. Nat. Genet..

[21] Aleksander S.A., Balhoff J., Carbon S., Cherry J.M., Drabkin H.J., Ebert D., Feuermann M., Gaudet P., Harris N.L., The Gene Ontology Consortium (2023). The Gene Ontology knowledgebase in 2023. Genetics.

[22] Danecek P., Auton A., Abecasis G., Albers C.A., Banks E., DePristo M.A., Handsaker R.E., Lunter G., Marth G.T., Sherry S.T. (2011). The variant call format and VCFtools. Bioinformatics.

[23] Alexander T.A., Machiela M.J. (2020). LDpop: An interactive online tool to calculate and visualize geographic LD patterns. BMC Bioinform..

[24] Machiela M.J., Chanock S.J. (2015). LDlink: A web-based application for exploring population-specific haplotype structure and linking correlated alleles of possible functional variants. Bioinformatics.

[25] Auton A., Brooks L.D., Durbin R.M., Garrison E.P., Kang H.M., Korbel J.O., Marchini J.L., McCarthy S., McVean G.A., Genomes Project Consortium (2015). 1000 Genomes Project Consortium. A global reference for human genetic variation. Nature.

[26] De Koning T.J., Snell K., Duran M., Berger R., Poll-The B.T., Surtees R. (2003). l-Serine in disease and development. Biochem. J..

[27] Szebenyi D.M.E., Musayev F.N., di Salvo M.L., Safo M.K., Schirch V. (2004). Serine Hydroxymethyltransferase: Role of Glu75 and Evidence that Serine Is Cleaved by a Retroaldol Mechanism. Biochemistry.

[28] Metcalf J.S., Dunlop R.A., Powell J.T., Banack S.A., Cox P.A. (2018). L-Serine: A Naturally-Occurring Amino Acid with Therapeutic Potential. Neurotox. Res..

[29] Tavirani M.R., Azodi M.Z., Rostami-Nejad M., Morravej H., Razzaghi Z., Okhovatian F., Rezaei-Tavirani M. (2020). Introducing Serine as Cardiovascular Disease Biomarker Candidate via Pathway Analysis. Galen Med. J..

[30] Mishra R.C., Tripathy S., Desai K.M., Quest D., Lu Y., Akhtar J., Gopalakrishnan V. (2008). Nitric Oxide Synthase Inhibition Promotes Endothelium-Dependent Vasodilatation and the Antihypertensive Effect of l -Serine. Hypertension.

[31] Nogal A., Alkis T., Lee Y., Kifer D., Hu J., Murphy R.A., Huang Z., Wang-Sattler R., Kastenmüler G., Linkohr B. (2023). Predictive metabolites for incident myocardial infarction: A two-step meta-analysis of individual patient data from six cohorts comprising 7897 individuals from the COnsortium of METabolomics Studies. Cardiovasc. Res..

[32] Yamakado M., Nagao K., Imaizumi A., Tani M., Toda A., Tanaka T., Jinzu H., Miyano H., Yamamoto H., Daimon T. (2015). Plasma Free Amino Acid Profiles Predict Four-Year Risk of Developing Diabetes, Metabolic Syndrome, Dyslipidemia and Hypertension in Japanese Population. Sci. Rep..

[33] Fan F., Liang Z., Liu Z., Sun P., Hu L., Jia J., Zhang Y., Li J. (2024). Association Between Serine Concentration and Coronary Heart Disease: A Case-Control Study. Int. J. Gen. Med..

[34] Hannun Y.A., Obeid L.M. (2018). Sphingolipids and their metabolism in physiology and disease. Nat. Rev. Mol. Cell Biol..

[35] Borodzicz-Jażdżyk S., Jażdżyk P., Łysik W., Cudnoch-Jȩdrzejewska A., Czarzasta K. (2022). Sphingolipid metabolism and signaling in cardiovascular diseases. Front. Cardiovasc. Med..

[36] Tabassum R., Ripatti S. (2021). Integrating lipidomics and genomics: Emerging tools to understand cardiovascular diseases. Cell. Mol. Life Sci..

[37] Chen H., Wang Z., Qin M., Zhang B., Lin L., Ma Q., Liu C., Chen X., Li H., Lai W. (2021). Comprehensive Metabolomics Identified the Prominent Role of Glycerophospholipid Metabolism in Coronary Artery Disease Progression. Front. Mol. Biosci..

[38] Dang V.T., Huang A., Zhong L.H., Shi Y., Werstuck G.H. (2016). Comprehensive Plasma Metabolomic Analyses of Atherosclerotic Progression Reveal Alterations in Glycerophospholipid and Sphingolipid Metabolism in Apolipoprotein E-deficient Mice. Sci. Rep..

[39] Clark A.G. (2004). The role of haplotypes in candidate gene studies. Genet. Epidemiol..

[40] Gilbert E., Shanmugam A., Cavalleri G.L. (2022). Revealing the recent demographic history of Europe via haplotype sharing in the UK Biobank. Proc. Natl. Acad. Sci. USA.

[41] Tada H., Fujino N., Hayashi K., Kawashiri M.-A., Takamura M. (2022). Human genetics and its impact on cardiovascular disease. J. Cardiol..

[42] Manna P., Jain S.K. (2015). Phosphatidylinositol-3,4,5-Triphosphate and Cellular Signaling: Implications for Obesity and Diabetes. Cell. Physiol. Biochem..

[43] Kächele M., Hennige A.M., Machann J., Hieronimus A., Lamprinou A., Machicao F., Schick F., Fritsche A., Stefan N., Nürnberg B. (2015). Variation in the Phosphoinositide 3-Kinase Gamma Gene Affects Plasma HDL-Cholesterol without Modification of Metabolic or Inflammatory Markers. PLoS ONE.

[44] Adams J.N., Raffield L.M., Freedman B.I., Langefeld C.D., Ng M.C., Carr J.J., Cox A.J., Bowden D.W. (2014). Analysis of common and coding variants with cardiovascular disease in the diabetes heart study. Cardiovasc. Diabetol..

[45] Palamiuc L., Ravi A., Emerling B.M. (2020). Phosphoinositides in autophagy: Current roles and future insights. FEBS J..

[46] Landrum M.J., Lee J.M., Riley G.R., Jang W., Rubinstein W.S., Church D.M., Maglott D.R. (2014). ClinVar: Public archive of relationships among sequence variation and human phenotype. Nucleic Acids Res..

[47] Xu X., Han L., Zhao G., Xue S., Gao Y., Xiao J., Zhang S., Chen P., Wu Z.-Y., Ding J. (2017). LRCH1 interferes with DOCK8-Cdc42-induced T cell migration and ameliorates experimental autoimmune encephalomyelitis. J. Exp. Med..

[48] Yang J., Yan B., Fan Y., Yang L., Zhao B., He X., Ma Q., Wang W., Bai L., Zhang F. (2019). Integrative analysis of transcriptome-wide association study and gene expression profiling identifies candidate genes associated with stroke. PeerJ.

[49] Malik R., Chauhan G., Traylor M., Sargurupremraj M., Okada Y., Mishra A., Rutten-Jacobs L., Giese A.-K., van der Laan S.W., Gretarsdottir S. (2018). Multiancestry genome-wide association study of 520,000 subjects identifies 32 loci associated with stroke and stroke subtypes. Nat. Genet..

[50] Bellomo T.R., Bone W.P., Chen B.Y., Gawronski K.A.B., Zhang D., Park J., Levin M., Tsao N., Klarin D., Lynch J. (2022). Multi-Trait Genome-Wide Association Study of Atherosclerosis Detects Novel Pleiotropic Loci. Front. Genet..

