# Supplementary material for: In Silico Systems Biology Approach for Prioritization of Candidate Genes Linked to Lipid Metabolism in the Context of Cardiovascular Disease Susceptibility in a Serbian Cohort
Source: Curr Issues Mol Biol. 2026 Jun 12;48(6):613. doi: 10.3390/cimb48060613 (PMC13298024; doi:10.3390/cimb48060613)
Supplement: Supplementary file 1 [file cimb-48-00613-s001.zip › Supplementary.pdf]

Table S1. The electron-ion interaction potential (EIIP) is used to encode amino acids.

Amino acid	EIIP [Ry]
Leu	0.0000
Ile	0.0000
Asn	0.0036
Gly	0.0050
Glu	0.0057
Val	0.0058
Pro	0.0198
His	0.0242
Lys	0.0371
Ala	0.0373
Tyr	0.0516
Trp	0.0548
Gln	0.0761
Met	0.0823
Ser	0.0829
Cys	0.0829
Thr	0.0941
Phe	0.0946
Arg	0.0959
Asp	0.1263

Table S2. The list of all interactors of the phosphoserine phosphatase (PSP) protein. Terms marked in green and with a (+) symbol are the five interactors with the highest change in the information spectrum method (ISM) signal amplitude. Proteins marked in red and with a (#) symbol are the five interactions with the lowest ISM signal amplitude.

Uniprot accession number	Uniprot protein name	Gene name	Amp(R49W)-Amp(WT)
Q9UH90 +	FBX40_HUMAN	*FBXO40*	0.29696409
O14939 +	PLD2_HUMAN	*PLD2*	0.256566101
Q32NB8 +	PGPS1_HUMAN	*PGS1*	0.239477513
Q8IYQ7 +	THNS1_HUMAN	*THNSL1*	0.233118296
P34896 +	GLYC_HUMAN	*SHMT1*	0.208853698
O15228	GNPAT_HUMAN	*GNPAT*	0.202746853
P35520	CBS_HUMAN	*CBS*	0.189898135
Q9ULN7	PNM8B_HUMAN	*PNMA8B*	0.1741045
P29353	SHC1_HUMAN	*SHC1*	0.128669578
Q9BVN2	RUSC1_HUMAN	*RUSC1*	0.128286854
O43175	SERA_HUMAN	*PHGDH*	0.122638882
Q9GZT4	SRR_HUMAN	*SRR*	0.080650852
O95749	GGPPS_HUMAN	*GGPS1*	0.062217904
P04406	G3P_HUMAN	*GAPDH*	0.055567778
Q9Y617	SERC_HUMAN	*PSAT1*	0.045270924
Q9HCL2	GPAT1_HUMAN	*GPAM*	0.043217019
Q5T2R2	DPS1_HUMAN	*PDSS1*	0.040982989
Q13393	PLD1_HUMAN	*PLD1*	0.037016441
Q86YH6	DLP1_HUMAN	*PDSS2*	0.036805262

P20132	SDHL_HUMAN	*SDS*	0.036403553
Q96GA7	SDSL_HUMAN	*SDSL*	0.034423673
O14556	G3PT_HUMAN	*GAPDHS*	0.032070834
Q86X67	NUD13_HUMAN	*NUDT13*	0.029833543
Q8N2A8	PLD6_HUMAN	*PLD6*	0.02718108
O15270	SPTC2_HUMAN	*SPTLC2*	0.014650095
O14735	CDIPT_HUMAN	*CDIPT*	0.010497722
Q9BQG2	NUD12_HUMAN	*NUDT12*	0.009140603
Q9UJA2	CRLS1_HUMAN	*CRLS1*	0.003473081
P21549	AGT1_HUMAN	*AGXT*	0.002522457
Q9BXS1	IDI2_HUMAN	*IDI2*	-0.014264067
Q13907	IDI1_HUMAN	*IDI1*	-0.063707295
O15269	SPTC1_HUMAN	*SPTLC1*	-0.066561169
Q6NUI2	GPAT2_HUMAN	*GPAT2*	-0.067853097
P14324 #	FPPS_HUMAN	*FDPS*	-0.090799305
P34897 #	GLYM_HUMAN	*SHMT2*	-0.205363183
Q9NUV7 #	SPTC3_HUMAN	*SPTLC3*	-0.234635509
Q86YJ6 #	THNS2_HUMAN	*THNSL2*	-0.256028761
Q8N2Y8 #	RUSC2_HUMAN	*RUSC2*	-0.816407101

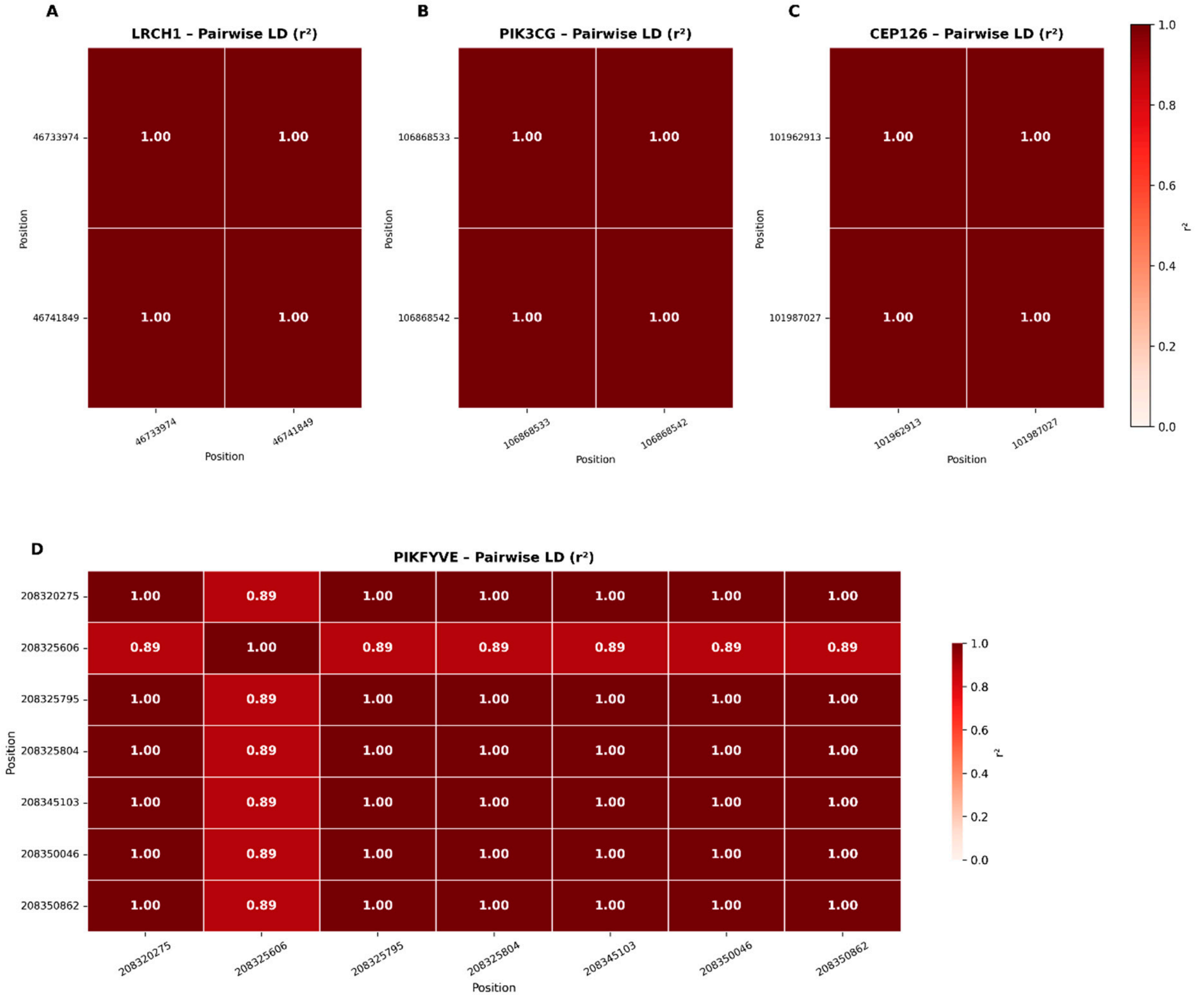

Figure S1 Pairwise LD between variants of A) LRCH1 gene, B) PIK3CG gene, C) CEP126 gene and D) *PIKFYVE* gene

Table S3 Pairwise LD calculation of gene variant pairs that are found in frequent combinations in the Serbian cohort, compared with the European populations of 1000 Genome Project

Population	N	Variant 1 Allele Freq	Variant 2 Allele Freq	R^2
LRCH1 **| Variant 1: chr13:46733974 | Variant 2: chr13:46741849**				
EUR	503	A: 0.3%, G: 99.7%	T: 0.3%, C: 99.7%	1.000
CEU	99	A: 0.0%, G: 100.0%	T: 0.0%, C: 100.0%	N/A
TSI	107	A: 0.93%, G: 99.07%	T: 0.93%, C: 99.07%	1.000
FIN	99	A: 0.0%, G: 100.0%	T: 0.0%, C: 100.0%	N/A
GBR	91	A: 0.0%, G: 100.0%	T: 0.0%, C: 100.0%	N/A
IBS	107	A: 0.47%, G: 99.53%	T: 0.47%, C: 99.53%	1.000
PIK3CG **| Variant 1: chr7:106868533 | Variant 2: chr7:106868542**				
EUR	503	A: 4.47%, G: 95.53%	T: 4.37%, C: 95.63%	0.977
CEU	99	A: 3.03%, G: 96.97%	T: 3.03%, C: 96.97%	1.000
TSI	107	A: 7.48%, G: 92.52%	T: 7.48%, C: 92.52%	1.000
FIN	99	A: 3.54%, G: 96.46%	T: 3.54%, C: 96.46%	1.000
GBR	91	A: 4.95%, G: 95.05%	T: 4.4%, C: 95.6%	0.884
IBS	107	A: 3.27%, G: 96.73%	T: 3.27%, C: 96.73%	1.000
CEP126 **| Variant 1: chr11:101962913 | Variant 2: chr11:101987027**				
EUR	503	C: 3.78%, T: 96.22%	G: 3.78%, A: 96.22%	1.000
CEU	99	C: 4.55%, T: 95.45%	G: 4.55%, A: 95.45%	1.000
TSI	107	C: 4.67%, T: 95.33%	G: 4.67%, A: 95.33%	1.000
FIN	99	C: 1.52%, T: 98.48%	G: 1.52%, A: 98.48%	1.000
GBR	91	C: 2.2%, T: 97.8%	G: 2.2%, A: 97.8%	1.000
IBS	107	C: 5.61%, T: 94.39%	G: 5.61%, A: 94.39%	1.000

PIKFYVE | Variant 1: chr2:208320275 | Variant 2: chr2:208350862				
EUR	503	C: 0.5%, T: 99.5%	A: 0.5%, G: 99.5%	1.000
CEU	99	C: 0.0%, T: 100.0%	A: 0.0%, G: 100.0%	N/A
TSI	107	C: 0.93%, T: 99.07%	A: 0.93%, G: 99.07%	1.000
FIN	99	C: 0.0%, T: 100.0%	A: 0.0%, G: 100.0%	N/A
GBR	91	C: 0.0%, T: 100.0%	A: 0.0%, G: 100.0%	N/A
IBS	107	C: 1.4%, T: 98.6%	A: 1.4%, G: 98.6%	1.000

Table S4 KEGG pathways of genes with variants found in a haplotype

Category	Term	Count	List Total	Pop Hits	Pop Total	P-Value	Benjamini	Fold Enrichment	Bonferroni	FDR	Fisher Exact	User Ids
KEGG_PATHWAY	Inositol phosphate metabolism	2	2	78	9496	0,00821	0,14	121,74	0,131	0,14	0,0000666	PIKFYVE,PIK3CG

Table S5 Gene Ontology biological processes of genes with variants found in a haplotype

Category	Term	Count	List Total	Pop Hits	Pop Total	P-Value	Benjamini	Fold Enrichment	Bonferroni	FDR	Fisher Exact	User Ids
GOTERM_BP_DIRECT	phosphatidylinositol-3-phosphate biosynthetic process	2	4	23	19512	0,00353	0,244	424,17	0,217	0,244	0,00000796	PIKFYVE,PIK3CG
GOTERM_BP_DIRECT	neutrophil chemotaxis	2	4	56	19512	0,00859	0,296	174,21	0,448	0,296	0,0000484	PIKFYVE,PIK3CG
